# Personal exposure levels to O_3_, NO_*x*_ and PM_10_ and the association to ambient levels in two Swedish cities

**DOI:** 10.1007/s10661-021-09447-7

**Published:** 2021-09-27

**Authors:** Susanna Lohman Haga, Annika Hagenbjörk, Anna-Carin Olin, Bertil Forsberg, Ingrid Liljelind, Hanne Krage Carlsen, Lars Modig

**Affiliations:** 1 Occupational and Environmental Medicine, School of Public Health and Community Medicine, Sahlgrenska Academy At University of Gothenburg, Gothenburg, Sweden; 2 Department of Public Health and Clinical Medicine, Section for Sustainable Health At Umeå University, Umeå, Sweden

**Keywords:** Air pollution, O_3_, NO_*x*_, PM_10_, Personal exposure

## Abstract

Exposure to air pollution is of great concern for public health although studies on the associations between exposure estimates and personal exposure are limited and somewhat inconsistent. The aim of this study was to quantify the associations between personal nitrogen oxides (NO_*x*_), ozone (O_3_) and particulate matter (PM_10_) exposure levels and ambient levels, and the impact of climate and time spent outdoors in two cities in Sweden. Subjects (*n* = 65) from two Swedish cities participated in the study. The study protocol included personal exposure measurements at three occasions, or waves. Personal exposure measurements were performed for NO_*x*_ and O_3_ for 24 h and PM_10_ for 24 h, and the participants kept an activity diary. Stationary monitoring stations provided hourly data of NO_*x*_, O_3_ and PM, as well as data on air temperature and relative humidity. Data were analysed using mixed linear models with the subject-id as a random effect and stationary exposure and covariates as fixed effects. Personal exposure levels of NO_*x*_, O_3_ and PM_10_ were significantly associated with levels measured at air pollution monitoring stations. The associations persisted after adjusting for temperature, relative humidity, city and wave, but the modelled estimates were slightly attenuated from 2.4% (95% CI 1.8–2.9) to 2.0% (0.97–2.94%) for NO_*x*_, from 3.7% (95% CI 3.1–4.4) to 2.1% (95% CI 1.1–2.9%) for O_3_ and from 2.6% (95% 0.9–4.2%) to 1.3% (95% CI − 1.5–4.0) for PM_10_. After adding covariates, the degree of explanation offered by the model (coefficient of determination, or *R*^2^) did not change for NO_*x*_ (0.64 to 0.63) but increased from 0.46 to 0.63 for O_3_, and from 0.38 to 0.43 for PM_10_. Personal exposure to NO_*x*_, O_3_ and PM has moderate to good association with levels measured at urban background sites. The results indicate that stationary measurements are valid as measure of exposure in environmental health risk assessments, especially if they can be refined using activity diaries and meteorological data. Approximately 50–70% of the variation of the personal exposure was explained by the stationary measurement, implying occurrence of misclassification in studies using more crude exposure metrics, potentially leading to underestimates of the effects of exposure to ambient air pollution.

**Supplementary information:**

The online version contains supplementary material available at 10.1007/s10661-021-09447-7.

## Background

Ambient air pollution is the largest environmental public health risk and is estimated to be responsible for approximately one in every ninth premature deaths annually worldwide (WHO, 2016). In average, 2.9 years of life expectancy are lost globally due to exposure to air pollutants (Lelieveld et al., 2020).

As air pollution is a complex mixture of different compounds, having both natural and anthropogenic origin, the ambient concentrations may vary depending on sources and local meteorological factors such as temperature, relative humidity, wind speed and direction (Grundström et al., 2015). In densely populated urban areas, traffic-related air pollutants at street level such as particulate matter (PM), nitrogen oxides (NO_*x*_) and ozone (O_3_) are of greatest concern as they are associated with severe both acute- and long-term health effects, particularly respiratory disease (WHO, 2016). Particulate matter (PM), complex mixtures of solid and liquid particles suspended in the air, can be of both anthropogenic and natural origin and are characterized by their size. Particles with an aerodynamic diameter smaller than 10 μm, PM_10_, mainly deposit in central airways but a small fraction will also reach the small airways (inner diameter < 2 mm), whereas fine particles smaller than 2.5 μm (PM_2.5_) reach further into the very peripheral airways and to a larger extent deposit in the transition zone, between conducting and acinar airways (Pinkerton KE, 2000). The most prominent sources of PM_10_ are local emissions related to traffic (Segersson et al., 2017), but PM_10_ levels are also influenced by long-range transport, which may account for up to 70% of the background levels in urban areas (Carlsen et al., 2020; Petit et al., 2019).

In urban areas, NO_*x*_ which is the common term for the nitrogen oxides NO and NO_2_ primarily originates from fossil fuel combustion in vehicles. In the presence of sunlight, NO_*x*_ reacts with volatile organic compounds whereby ground-level O_3_, a powerful oxidant and airway irritant, is formed. In urban areas, high levels of O_3_ occur due to influx of long-range transport and locally emitted precursor gases, mainly NO_*x*_ (Hagenbjörk et al., 2017). O_3_ tends to peak in spring at high latitudes due to meteorological variation (Boleti et al., 2020).

Although air pollution concentrations measured at stationary monitoring stations are not very representative of personal exposure (Johannesson et al., 2007), exposure models such as dispersion models validated against stationary measurements are standard exposure assessment methods in studies of health risks in humans (Dias & Tchepel, 2018). Misclassification of exposure leads to reduced accuracy (Berkson error), or underestimates of health risk in epidemiological studies (Sheppard et al., 2012), which has been observed for a number of respiratory health outcomes (Hart et al., 2015; Van Roosbroeck et al., 2008; Weichenthal et al., 2015).

The current work is part of a larger study designed to investigate the effects of air pollution and birch pollen exposure in individuals with birch allergy and asthma and healthy controls at different seasons of the year.

The aim of this study was to quantify the agreement between urban background and personal exposure of NO_*x*_, O_3_ and PM_10_ to increase our knowledge of monitored concentrations at urban background stations as substitutes for personal exposure in population studies. Another aim was to estimate to what extent factors such as geographic location, meteorology and self-reported exposure (i.e., time spent outdoor in traffic) affect the associations.

## Methods

### Study protocol

Two Swedish cities were included in the study, Gothenburg in the south (57° N) and Umeå in the north (63° N), and adult individuals were invited to participate in the study. In Umeå, participants were recruited from the clinical part of the GA_2_LEN (Global Allergy and Asthma European Network) study (Jarvis et al., 2012) and in Gothenburg by an advertisement at the University of Gothenburg and in a local newspaper. A total of 65 individuals aged 27–76 years and a mean age 48.7 years were included in the study (Table 1).

**Table 1:** Descriptive statistics for the study population

	All participants	Gothenburg	Umeå
	*n* = 65	*n* = 37	*n* = 28
Male sex	30 (46%)	17 (46%)	13 (46%)
Height (cm)	173.5 ± 8.8	172.2 ± 8.7	175.2 ± 8.7
Age (years)	48.7 ± 13.6	47.8 ± 15.1	49.9 ± 11.6

**Fig. 1:**
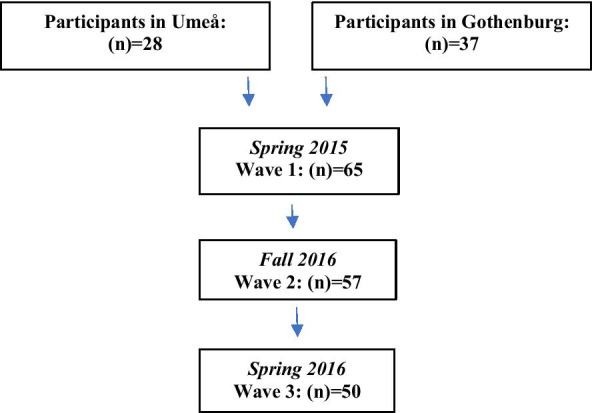
A total of 65 participants were recruited to participate in the study. A few participants dropped out of the study due to withdrawal, medical issues or moving. A total of 50 participants completed all three measuring periods (waves 1, 2 and 3)

The study protocol included a 10-day personal exposure measuring period at three separate occasions for each subject; the first occasion, wave 1, took place during April/May 2015. The second wave (wave 2) in November 2015 and the third wave (wave 3) in April/May 2016. The participants filled out an activity diary throughout the sampling period in which time spent in different environments such as (1) indoors, (2) outdoors in dense traffic and (3) outdoors (not in traffic) was documented. Following each sampling period, the participants underwent a thorough clinical examination. After the first measuring period (wave 1), a few participants dropped out of the study due to withdrawal, medical issues or moving. A total of 50 participants completed all three measuring periods, waves 1, 2 and 3 (Fig. 1).

Out of the initial 65 participants, personal measurements of all three pollutants (NO_*x*_, O_3_ and PM_10_) were obtained from 61 subjects in wave 1, from 54 subjects in wave 2 and from 44 subjects in wave 3. Also, there were 48 subjects who had three repeated (e.g., measured in all three waves) measurements of O_3_, 47 subjects who had three repeated NO_*x*_ measurements and 45 subjects with three completed measurements of PM_10_ in all three waves. The reasons for non-complete data at wave 1 are lost samplers, and a few participants deciding to withdraw from certain measurements. From wave 1 to wave 2 and 3, a few participants dropped out due to medical issues unrelated to the study, finding the protocol and the measurement equipment unhandy, or due to relocating to another city. Sixty-four participants filled out the activity diary for wave 1, but only 52 had valid replies. For waves 2 and 3, 47 and 44 valid replies were received, respectively.

### Personal exposure measurements, samplers and chemical analysis

#### ***NO***_***x***_*** and O***_***3***_

Passive samplers for NO_*x*_ and O_3_ were attached to a fabric cord resembling a necklace and placed as close to the breathing zone as possible. Participants were instructed to wear the samplers all day and place them by the bed when sleeping. In the case of precipitation, they were told to shield the samplers from getting wet.

NO_*x*_ and O_3_ were measured with the Ogawa diffusive sampler (Ogawa & Company, Pompano Beach, FL, USA) as 10-day averages of each compound. The Ogawa sampler is cylindrical and has a two-ended design with a diffusion barrier, and a coated filter between two stainless screens on each side. NO_*x*_ was collected on one Ogawa badge provided with a filter coated with triethanolamine (TEA) and an oxidizing substance, 2-phenyl-4,4,5,5-tetramethylimidazoline-1-oxyl-3-oxide (PTIO) added to oxidize NO to NO_2_. The nitrite content of the collection filter was determined by ion chromatography as described previously (Hagenbjörk-Gustafsson et al., 2010). The detection limit was 0.12 μg/m^3^ for a 10-day sampling period of NO_*x*_.

For O_3_ measurements, another Ogawa badge was used holding collection filters coated with a nitrite-based solution. O_3_ oxidizes nitrite to nitrate on the filter and the nitrate content of the filter was after extraction determined by ion chromatography according to a modified method of the standard operation procedure, published by Ogawa (www.ogawausa.com). The nitrate concentration was used to calculate the amount of O_3_ on each filter. The detection limit was 0.86 μg/m^3^ for a 10-day sampling period of O_3_. In cases where O_3_ was measured below this level (*n* = 3), it was substituted with the detection limit (0.86 μg) divided by two (Schisterman et al., 2006).

The coated filters for NO_*x*_ and O_3_ were supplied by the manufacturer (Ogawa, USA). All samples were prepared and analysed at the division of Occupational and Environmental Medicine, Umeå University, Umeå.

#### ***PM***_***10***_

An active sampling of PM_10_ was performed 24 h prior to the clinical visit. Each participant was handed out a backpack equipped with an AirChek® XR5000 personal air sampling pump (SKC Inc., Eighty Four, PA, USA), mounted with a single-stage Personal Modular Impactor (PMI) sampler for PM_10_ collection (SKC Inc., Eighty Four, PA, USA) and an airflow of 3.0 L/min. The airflow was calibrated prior to and at the end of the 24-h sampling period. The PMI sampler was mounted with a 25-mm pre-oiled impaction disc on top of the filter cassette with a 2-μm pre-weighted Millipore PTFE collection filter for gravimetric analysis at Occupational and Environmental Medicine, School of Public Health and Community Medicine at University of Gothenburg, Gothenburg, Sweden.

### Stationary measurements

#### NO_x_,O_3_ and PM

In Gothenburg, the local environment department provided hourly data on NO_*x*_, PM_10_ and O_3_ concentrations. The measurements were performed at the main measurement station in Gothenburg, “Femman” situated at a rooftop (height 27 m) in central Gothenburg (57° 42.52ʹ N, 11° 58.23ʹ E). NO_*x*_ was measured with a chemiluminescence detector (Model T200 NO/NO_2_/ NO_*x*_ Analyzer, Teledyne API, San Diego, USA).

PM_10_ was measured by using the tapered element oscillating microbalance technique (Thermo Scientific^TM^1405 TEOM™ Continuous Ambient Particulate Monitor, Thermo Fischer Scientific, Waltham, USA). O_3_ measurements were carried out by using UV photometry (Monitor Labs, O3 ML 9811, Monitor Labs, Karlsruhe, Germany).

In Umeå, NO_*x*_ and O_3_ were measured at the former municipality background station at a rooftop (height 20 m) located in the city centre of Umeå (63° 79.47ʹ N, 20° 29.18ʹ E). NO_*x*_ was measured using a chemiluminescence analyser (Monitor Labs model 9841, Monitor Europe, Cheltenham, UK). Hourly data of O_3_ was provided by a UV photometer (Monitor Labs model 9810, Monitor Europe, Cheltenham, UK). Measurements of PM_10_ were only provided at a street station in central Umeå and therefore not included in the study; however, PM_2.5_ was measured at an urban background station (preschool Uven, 63° 82.09 N, 20° 28.96 E) about 1 km from the city centre. Twenty-four-hourly data of PM_2.5_ was provided from IVL, Swedish Environmental Research Institute, and was measured by a standard gravimetric measurement method (Leckel SEQ47/50, Leckel Ingenieurbüro GMBH, Berlin, Germany).

An annual mean background exposure level of PM_2.5_ was modelled in the Swedish Clean Air and Climate project with dispersion modelling (Segersson et al., 2017) and matched with the participants’ residential address coordinates.

#### Meteorological data

The Swedish Meteorological and Hydrological Institute provides hourly data on air temperature, relative humidity and wind speed and direction measured centrally in Gothenburg (57° 71.56 N, 11° 99.24 E) and at Umeå airport (63° 79.47 N, 20°29.18 E), approximately 4 km from the city centre.

#### Statistical methods

For each participant, the time window of personal exposure to each pollutant (of 24 h or 10 days) were matched with data from the stationary monitoring station for the corresponding time. For personal and stationary measurements, mean and standard deviation were calculated. Median and interquartile range (IQR) were calculated for self-reported time spent in- and outdoors (self-reported exposure) as these variables were strongly skewed. Individuals who had personal and stationary measurements from at least one study wave were included in the study.

The data were analysed using mixed linear models (Delfino et al., 2006).

$$\mathrm{log}(\mathrm{Y}) = {\beta }_{1}\mathrm{Stat}\_\mathrm{exp }+ {\beta }_{2}\mathrm{Time}+ {\beta }_{3}\mathrm{Temp }+ {\beta }_{4}\mathrm{RH }+ {\beta }_{5}\mathrm{City }+ {\beta }_{6}\mathrm{Wave }+ (1 |\mathrm{ ID})$$

where *Y*, the dependent variable, is personal-measured exposure, Stat_exp is stationary monitor measured exposure, Time is time spent outside in dense traffic, Temp is temperature at the same interval as the main exposure, RH is relative humidity at the same interval as the main exposure, City denotes the study location and Wave is the study season. ID is the personal identification number of every individual inserted as a random effect. To test for further random effects, study wave and city were also tested as random effects in the models, but the model fits were not improved, and those variables were entered as covariates.

The dependent variables for each model were transformed with natural logarithms to approach normality.

First, the personal exposure was modelled as a function of the levels measured at the stationary monitoring site of the corresponding pollutant, then the covariates weather and temperature were added, then city and study wave. Study wave was treated first as a three-level variable for the three waves, then as a two-level variable to indicate spring season (waves 1 and 3) or fall season (wave 2). In a separate analysis, covariates “time spent in traffic,” “time outdoors not in traffic,” “total time outdoors” and “time indoors” were added to models to estimate their individual effects. As a sensitivity analysis, individuals with birch allergy and asthma were analysed separately to determine if any eventual exposure avoidance (less self-reported time spent in traffic) influences the association between personal and stationary levels.

To quantify the variance explained by the models the conditional coefficient of determination (*R*^2^) was determined for the models (Nakagawa & Schielzeth, 2013). The analysis was performed with R studio, and the “lme4” package (Bates et al., 2014). The level of significance was set to *p* < 0.05.

## Results

### Personal and stationary levels of NO_x_ and O_3_ and PM_10_

NO_*x*_ levels were higher in wave 2, i.e., the fall season, for both personal and stationary measurements at both locations. For O_3_, a strong seasonal variation was seen in Umeå with highest levels in spring seasons (waves 1 and 3). This seasonal variation was not as clearly seen in Gothenburg, even though the levels were highest in the first wave (spring) compared to waves 2 and 3. PM_10_ levels had only minor seasonal variations.

**Fig. 2:**
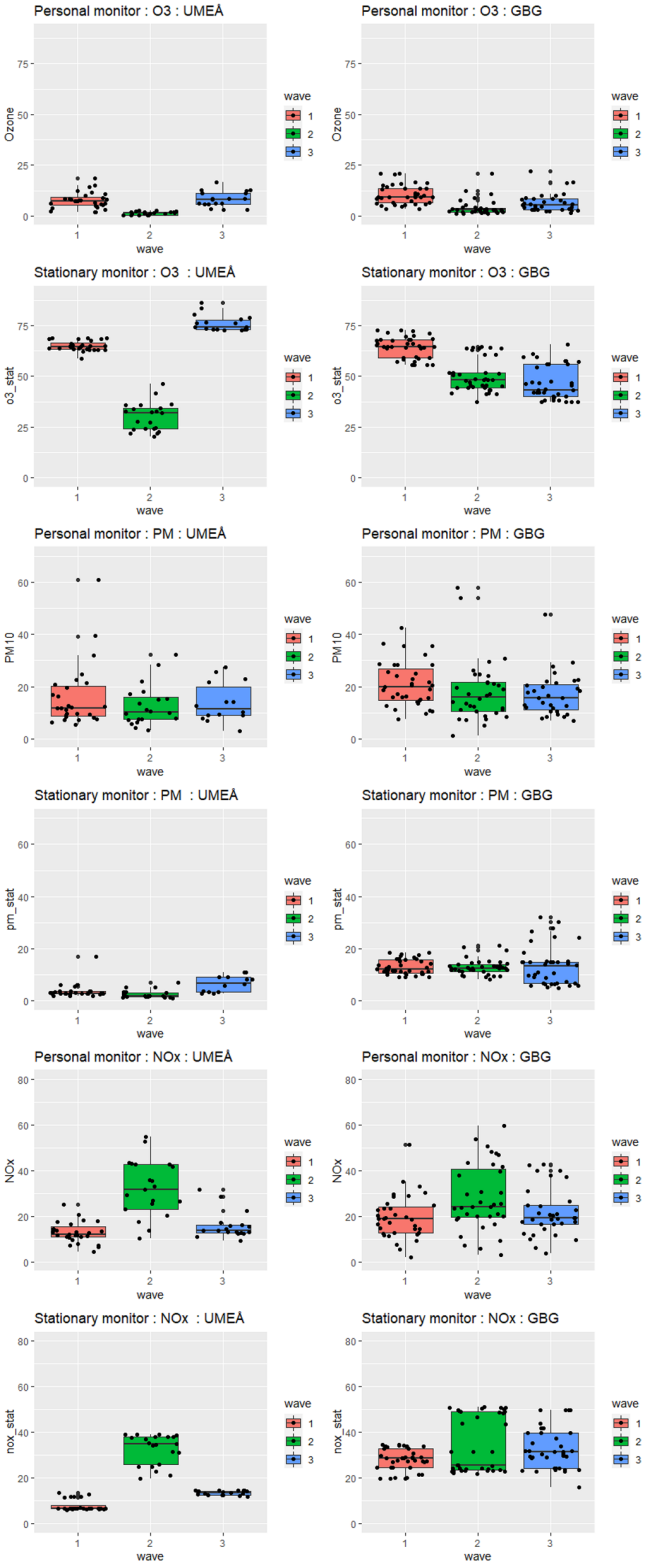
Personal and stationary exposure. One PM outlier is omitted. Stationary PM of Umeå is PM_2.5_

Comparing stationary and personal measurements of air pollution levels, in general, the personal measurements indicated lower exposure than the stationary measurements. The differences were most pronounced for O_3_, with stationary levels of 53.7 ± 10.6 and 56.9 ± 19.4 μg/m^3^, compared to personal levels of 7.2 ± 5.2 and 5.9 ± 4.5 μg/m^3^ in Gothenburg and Umeå, respectively. The personal NO_*x*_ exposure levels in Umeå as well as the personal PM_10_ exposure levels in Gothenburg was however an exception, as the levels were similar or higher than levels registered at the stationary monitoring stations (Table 2 and Fig. 2).

**Table 2:** Descriptive statistics (mean ± standard deviation) for exposure variables for the study population in the relevant exposure interval

	Wave	*N*	Gothenburg (*n* = 37)		Umeå (*n* = 28)		Personal	Stationary
			Personal	Stationary	Personal	Stationary	*p*	*p*
NO_*x*_ (μg/m^3^) (10 days)	All	170	23.1 ± 12.2	31.8 ± 9.5	20.8 ± 14.6	17.1 ± 11.5	0.29	< 0.001
	1	63	19.5 ± 9.6	27.9 ± 4.8	15.7 ± 15.7	8.0 ± 2.5	0.26	< 0.001
	2	56	28.0 ± 14.6	34.5 ± 12.3	31.7 ± 12.4	32.7 ± 6.6	0.32	0.47
	3	51	21.5 ± 10.5	32.9 ± 8.9	15.9 ± 5.9	13.5 ± 0.9	0.02	< 0.001
O_3_ (μg/m^3^) (10 days)	All	171	7.2 ± 5.2	53.7 ± 10.6	5.9 ± 4.5	56.9 ± 19.4	0.10	< 0.001
	1	65	10.5 ± 4.8	64.3 ± 5.3	7.8 ± 4.0	64.9 ± 2.5	0.01	0.59
	2	56	4.0 ± 3.9	50.3 ± 11.5	1.4 ± 0.8	30.0 ± 7.1	< 0.001	< 0.001
	3	50	6.7 ± 4.6	46.6 ± 4.6	8.3 ± 3.8	76.1 ± 3.8	0.19	< 0.001
PM_10_ (μg/m^3^) (24 h)	All	164	23.1 ± 28.7	13.2 ± 5.0	14.4 ± 9.9	3.9 ± 2.8*	0.006	< 0.001
	1	63	23.1 ± 15.6	13.1 ± 3.0	16.1 ± 12.1	3.7 ± 2.9*	0.047	< 0.001
	2	55	22.6 ± 26.8	12.8 ± 3.0	12.6 ± 7.8	2.6 ± 1.6*	0.06	< 0.001
	3	46	24.0 ± 40.5	13.6 ± 7.6	13.9 ± 7.6	6.6 ± 3.1*	0.18	< 0.001

*Stationary measurements refer to PM_2.5_ in Umeå.

*p*-values from a *t*-test

### *Modelled data of PM*_*2.5*_* at residential address*

The modelled background exposure to PM_2.5_ at the residential address was lower in Umeå than in Gothenburg (mean 0.66 μg/m^3^ and 6.6 μg/m^3^, *p* < 0.001). However, using modelled background data to adjust for location did not improve the model fits or change the association between personal and stationary monitor measured PM (data not shown).

### Activity diary

Participants in Gothenburg reported spending significantly more time in dense traffic than those in Umeå at all study waves (median 94, 64 and 86 min in Gothenburg vs 52, 46 and 51 min in Umeå), whereas there were only small non-systematic differences in the reported time spent outdoors outside dense traffic, and indoors, between the two study locations. During the spring season (waves 1 and 3), the study participants reported spending more time outdoors than in fall season (wave 2) (Table 3).

**Table 3:** Self-reported time spent outdoors in dense traffic, outdoors but not in dense traffic, and indoors as average per day in the 10-day sampling period and on the last day (24 h) before the clinical visit

	Wave			*N*		Göteborg	Umeå		p*	
						Median ± IQR	Median ± IQR			
**Outdoors in dense traffic (min)**										
10 days		1		50	94.3 ± 79.1			51.9 ± 55.9		**0.002**
		2		45	64.5 ± 71.3			46.2 ± 45.0		**0.04**
		3		44	85.8 ± 68.0			51.3 ± 76.5		**0.009**
24 h		1		52	60.0 ± 90.0			5.0 ± 60.0		**0.004**
		2		47	60.0 ± 90.0			30.0 ± 50.0		**0.028**
		3		44	50.0 ± 67.5			30.0 ± 60.0		0.283
**Outdoors—not dense traffic (min)**										
10 days		1	49		72.0 ± 51.8			99.0 ± 95.3		0.105
		2	43		49.2 ± 48.8			37.5 ± 84.0		0.392
		3	40		81.5 ± 58.5			125.5 ± 83.3		0.125
24 h		1	50		30.0 ± 72.5			30.0 ± 52.5		0355
		2	44		30.0 ± 52.5			0.0 ± 37.5		**0.066**
		3	40		60.0 ± 106.3			60.0 ± 120.0		0.955
**Indoors (h)**										
10 days		1	48		20.6 ± 2.9			20.8 ± 2.0		0.992
		2	44		21.4 ± 1.8			21.9 ± 2.0		0.200
		3	40		20.6 ± 1.8			20.7 ± 1.4		0.989
24 h		1	49		21.0 ± 3.3			17.5 ± 11.1		0.189
		2	44		21.0 ± 3.9			21.0 ± 6.5		0.972
		3	41		19.0 ± 10.5			19.5 ± 7.2		0.841

*p-value extracted from unpaired, two-sided Wilcoxon signed-rank test

### Regression analysis

A likelihood ratio test revealed that a two-level variable of “study wave” (non-pollen season versus pollen season) was the best fit for NO_*x*_ and O_3_ (*p* < 0.05). However, for PM, three levels (one for each study wave) produced a better model fit (*p* < 0.05), and better Akaike’s information criteria (AIC), but worse Bayes information criteria (BIC) (Table S2), so the three-level variable was selected for the remaining analysis of PM.

In mixed models, the levels of the pollutant measured at the urban background stations were significantly associated with the log-transformed personal exposure levels of the same pollutant. After adjusting for temperature, relative humidity, city and spring seasons (waves 1 and 3) vs fall (wave 2), the regression slopes were less steep but remained statistically significant for NO_*x*_ and O_3_. The degree of explanation (*R*^2^) of the unadjusted models was moderate at 0.38 for PM_10_, marginally higher for O_3_ at 0.46 and 0.63 for NO_*x*_. However, the degree of explanation increased after introducing covariates for O_3_ and PM_10_ but was nearly unchanged for NO_*x*_ (Table 4). Relative humidity was statistically significantly associated with NO_*x*_ and O_3_ before adjusting for city and wave. City was statistically significantly associated with O_3_. For O_3_ and PM_10_ exposure, there was a negative association with spring season. The proportion of variation explained by the models (*R*^2^) was highest for the NO_*x*_ model at 0.64 for the fully adjusted model. For O_3_, the fully had adjusted model *R*^2^ was 0.63, and for PM_10_ it was 0.43 (Table 4) indicating a modest degree of explanation.

**Table 4:** Associations between personal and stationary monitor measured from unadjusted and adjusted mixed models with personal exposure as dependent variable, stationary measurements in covariates as predictors, and ID as a random effect

Personal monitor	*N*	Stationary measurement (μg/m^3^)	Temperature (°C)	Relative humidity (%)	City	Wave^a^	*R*^2b^
		**% (95% CI)**	**% (95% CI)**	**% (95% CI)**	**% (95% CI)**	**% (95% CI)**	
**NO**_***x***_ (μg/m^3^) 10 days	**170**	**2.4 (1.8–2.9)**	-		-	-	0.63
		**2.1 (1.4–2.7)**	0.2 (− 2.3–2.8)	**1.1 (0.3–1.8)**	-	-	0.63
		**2.0 (1.0–2.9)**	0.1 (− 3.5–3.1)	12.0 (− 15.3–54.8)	12.0 (− 15.3–39.3)	18.4 (− 18.0–54.8)	0.64
**O**_**3**_** (μg/m**^3^**)** 10 days	**171**	**3.7 (3.1–4.4)**	-	-	-	-	0.46
		**2.2 (1.3–3.1)**	1.3 (− 2.6–5.3)	** − 3.3 (− 4.5– − 2.1)**	-	-	0.56
		**2.0 (1.1–2.9)**	1.4 (− 2.7–5.4)	0.6 (− 1.5–2.6)	** − 37.5 (− 64.2– − 10.8)**	** − 89.8 (− 129.8– − 49.9)**	0.63
**PM**_**10**_** (μg/m**^3^**)** 24 h	**163**	**2.6 (0.9–4.2)**	-	**-**	**-**	**-**	0.38
		2.0 (− 0.1–4.0)	1.2 (− 1.3–3.6)	0.1 (− 0.8–1.0)	-	**-**	0.39
		1.3 (− 1.5–4.0)	1.0 (− 2.0–3.9)	0.4 (− 0.7–1.4)	− 31.5 (− 64.2–1.2)	** − 24.0 (− 49.6–1.7)**^**c**^	0.43

Adjusted model example: model <  − lmer(log(PM) ~ pm_stat + temperature + relative humidity + city + wave + (1 | ID), data = long_data). Results are given as percentage change per unit change in the predictor variable. The outcome variable was log-transformed with natural logarithm

^a^Effect of fall season relative to spring seasons (reference)

^b^Condition coefficient of determination adapted for mixed models

^c^Coefficient for wave 3 is − 21.1 (− 42.1– − 0.2)

Comparing the influence of different metrics of self-reported exposure (time spent outdoors or in traffic) on the associations between personal and stationary exposure levels (Table 5), time in traffic and total time outdoors were positively associated with the personal exposure for NO_*x*_, and the estimate of the stationary measurement station was slightly lower than the main analysis (Table 4). For O_3_, time outdoors not in traffic and total time outdoors were positively associated with personal exposure, and time spent indoors was negatively associated with personal exposure. Again, the estimated association with the stationary measurements was lower than in the main analysis. For PM_10_, no self-reported exposure was associated with personal exposure (Table 4). However, for PM_10_, the coefficient of association was increased in models adjusted for time spent outdoors or in traffic, but only reached statistical significance in the model adjusted for time spent in traffic (Table 5).

**Table 5:** Associations between personal exposure and stationary pollution measures adjusted for activity log-reported time outdoors in traffic, out of traffic, total time outdoors and time indoors from mixed models with exposure and covariates as predictors and ID as a random effect

Personal monitor	*N*	Stationary measurement (μg/m3)	Time in traffic^a^ (min)	Time outdoors not in traffic (min)	Total time outdoors (min)	Time indoors (h)	*R*^2b^
		**% (95% CI)**	**% (95% CI)**	**% (95% CI)**	**% (95% CI)**	**% (95% CI)**	
**NO**_***x***_** (μg/m**^**3**^**)** 10 days	**138**	**1.9 (0.8–3.0)**	**0.2 (0.0–0.3)**		-		0.64
	**131**	**1.6 (0.4–2.8)**		0.1 (− 0.0–0.1)			0.60
	**128**	**1.4 (0.3–2.5)**			**0.1 (0.0–0.2)**		0.64
	**131**	**1.8 (0.6–3.0)**				− 2.4 (− 5.6–0.9)	0.60
**O**_**3**_** (μg/m**^**3**^**)** 10 days	**138**	**1.5 (0.6–2.5)**	0.2 (− 0.0–0.4)		-		0.66
	**131**	**1.4 (0.4–2.4)**		**0.2 (0.1–0.3)**			0.66
	**128**	**1.4 (0.4–2.3)**			**0.2 (0.1–0.3)**		0.70
	**131**	**1.2 (0.2–2.2)**	-	-	-	** − 4.5 (− 8.4– − 0.5)**	0.66
**PM**_**10**_** (μg/m**^**3**^**)** 24 h	**136**	**3.0 (0.0–6.1)**	0.0 (− 0.1–0.2)	-		-	0.48
	**127**	3.0 (− 0.3–6.2)		0.1 (− 0.0–0.1)		-	0.42
	**126**	3.1 (− 0.2–6.3)			0.1 (− 0.0–0.1)		0.44
	**126**	3.1 (− 0.1–6.3)				− 0.0 (− 1.6–1.6)	0.49

^a^Self-reported time spent outdoors in dense traffic the last 10 days for NO_*x*_ and O_3_, and the last 24 h for PM_10_

^b^Conditional coefficient of determination adapted for mixed models

## Discussion

In this panel study 65 participating individuals from two Swedish cities with substantially different background pollution levels and meteorology, reported their daily activities and simultaneously had their personal exposure monitored for up to three measurement periods. Stationary measures of exposure to NO_*x*_, O_3_ and PM_10_ were statistically significantly associated with personal exposure in unadjusted, mixed models with individual as random effects (Table 4). After adding covariates, such as meteorological variables, city and wave, stationary PM was no longer statistically significantly associated with personal PM, but for all three outcomes, the model fits were improved after adding covariates as indicated by increases in *R*^2^. The fully adjusted models of NO_*x*_ and O_3_ explained more than 50% of the variation in the personal exposure, although the number of observations decreased due to dropout and non-participation, especially for the self-reported exposure in the activity diary. In mixed models, the levels of the pollutant measured at the urban background stations were significantly associated with the log-transformed personal exposure levels of the same pollutant.

Participants in Gothenburg generally reported spending more time outdoors in dense traffic which is logical as Gothenburg is a larger city with substantially more dense traffic compared to Umeå (Carlsen et al., 2017) (Table 3). In general, people spend most of their time indoors. In the current study, the participants reported spending an average of around 21 h indoors in both spring (wave 1 and 3) and winter (wave 2).

The time outdoors in dense traffic was significantly associated with personal NO_*x*_ exposure and as expected, contributed significantly to the individuals’ exposure (Table 5). For O_3_, total time spent outdoors, time spent outdoors not in traffic, and time inside were significantly associated with personal exposure to O_3_ (time spent indoors was negatively correlated), whereas the association with time spent in dense traffic was also positive, it did not reach statistical significance, possibly because of the complex chemical reactivity pattern of O_3_ in dense traffic.

Time spent indoors was negatively correlated with all personal exposures, although it only reached statistical significance for O_3_. Also, for PM_10_, the association between personal exposure and stationary measurements were stronger after adjusting for time spent in dense traffic, although the association for time spent in traffic did not reach statistical significance, perhaps because time spend in dense traffic strongly influence the personal exposure measurements (Table 5).

To improve the adjustment for location, the models were adjusted for modelled annual background levels of PM_2.5_ instead of city. However, this variable did not improve the model fit and did not modify the effect of the stationary PM_10_ exposure (Table S3) in the short term. As Gothenburg is in the southern part of Sweden, a larger proportion of air pollutants is due to long-range transport from more southern parts of Europe compared to Umeå in the northern part of Sweden. However, air pollutants are generated both locally and transported some distances with the wind but have little within-city gradient and are thus not likely to influence the results of this study. Furthermore, because of its reactivity, NO_*x*_ decays in the atmosphere within days before it can be subjected to long-range transport away from the source. The size of the proportion of PM contributed from long-range transport is a matter of debate and wide ranges have been reported. Johannesson et al. (2007) observed associations between 24 h of urban background and personal levels of PM_2.5_ particles with a correlation coefficient of 0.61 (Spearman) but spending time outdoors was only a predictor for the Fe-trace element. In a multi-centre study in heterogeneous environments the authors compared land use regression (LUR)-based exposure with personal exposure and found that LUR predicted personal exposure to soot and NO_2_, in some sites with *R*^2^ from 0.35 to 0.44 (Montagne et al., 2013) For PM_2.5_ and NO_*x*_, there were no significant correlations. Measuring in elderly subjects during spring, summer and winter, it was found that LUR model-predicted O_3_ and PM_2.5_ showed moderately associations with personal exposure levels, whereas model-predicted NO_2_ was not associated with personal NO_2_ (Sahsuvaroglu et al., 2009). Thus, there is no consensus regarding personal exposure to air pollutants based on stationary measurements, and therefore, until now, it has also been difficult to sort out if certain exposures are more harmful, which to some extent can be explained by rough exposure assessments that will blur the effects of specific exposures. In studies that aimed to quantify the effect of measurement error, it was found that risk estimates increased after adjustment for measurement error (Hart et al., 2015). This important point will be addressed in future analysis of the collected data as no health risks were addressed in the current study.

### Strengths and limitations

The study design with thorough sampling and repeated measures on the same individual during three monitoring waves as well as parallel self-reported activity ensures that our data has high internal validity. The study was performed using an identical study protocol and identical equipment for measurements of personal exposure, in two distinct geographical locations with different meteorology and background exposure, which ensured that the data had good variability.

Due to various reasons, among them, a comprehensive study protocol and a few lost samplers, not all subjects were included in all measurements in all study waves; however, a comparison of the demographic characteristics and exposure of the individuals who did not complete all exposure measurements (*n* = 18) versus those who did (*n* = 47) found no statistically significant differences.

## Conclusion

In this study, there were moderate to good associations between personal and stationary measurements of NO_*x*_, O_3_ and PM, which were strengthened by data on meteorology and covariates. The absolute levels of O_3_ showed substantially lower personal exposure levels compared to stationary levels. The addition of self-reported time spent in traffic improved the model in the case of NO_*x*_ and O_3_, whereas for PM_10_, self-reported time spent in traffic or outdoors was not significant, perhaps reflecting the importance of exposure other than traffic, e.g., occupational exposure.

The observed results support that stationary measurements are valid as a measure of exposure in environmental health risk assessments, especially if they can be refined using activity diaries and measures of meteorology. Nevertheless, only 50–70% of the variation in the personal exposure was explained by the stationary measurement, implying the occurrence of misclassification in studies using more crude exposure metrics, potentially leading to underestimates of the effects of exposure to ambient air pollution.

## Supplementary Information

Below is the link to the electronic supplementary material.

Supplementary file1 (PDF 14 KB)

Supplementary file2 (PDF 68 KB)

## Data Availability

The datasets used and/or analysed during the current study are available from the corresponding author on request.

## Abbreviations

PM: Particulate matter
PM_2.5_: Particulate matter ≤ 2.5 µm in diameter
PM_10_: Particulate matter ≤ 10 µm in diameter
O_3_: Ozone
NO_*x*_: Nitrogen oxides
NO_2_: Nitrogen dioxide
SD: Standard deviation
IQR: Interquartile range
RH: Relative humidity
*R*^2^: Coefficient of determination
LUR: Land use regression
AIC: Akaike’s information criteria
BIC: Bayes information criteria
