# Supplementary material for: Personal exposure levels to O3, NOx and PM10 and the association to ambient levels in two Swedish cities
Source: Environ Monit Assess. 2021 Sep 27;193(10):674. doi: 10.1007/s10661-021-09447-7 (PMC8476356; doi:10.1007/s10661-021-09447-7)
Supplement: Supplementary file 1 — Supplementary file1 (PDF 14 KB) [file 10661_2021_9447_MOESM1_ESM.pdf]

		Personal			Central monitor		
		NO_X	ozone	PM	NO_X	ozone	PM
Personal	NO_X	1	-.23**	.18*	.37***	-.34***	.02
	Ozone		1	.05	-.21**	.46***	.13
	PM			1	.21**	-.00	.12
Central monitor	NO_X				1	-.56***	.38***
	Ozone					1	0.10
	PM						1

*p<0.05, **p<0.01, ***p<0.001, stationary monitor PM is $PM_{2.5}$ in Umeå, PM_{10} in Gothenburg.
