# Supplementary material for: Personal exposure levels to O3, NOx and PM10 and the association to ambient levels in two Swedish cities
Source: Environ Monit Assess. 2021 Sep 27;193(10):674. doi: 10.1007/s10661-021-09447-7 (PMC8476356; doi:10.1007/s10661-021-09447-7)
Supplement: Supplementary file 2 — Supplementary file2 (PDF 68 KB) [file 10661_2021_9447_MOESM2_ESM.pdf]

	Wave adjustment	Stationary pollutant	AIC*	BIC*	R^2	P*
		B (se)				-
NO_X	No wave	2.4 (0.3)	243	255	0.63	(reference)
	2 **	1.7 (0.0)	233	249	0.65	<0.001
	3 ***	1.7 (0.4)	235	254	0.64	0.47
O_3	No wave	3.7 (0.4)	371	383	0.46	(reference)
	2	2.1 (0.4)	327	343	0.63	<0.001
	3	2.0 (0.4)	327	346	0.63	0.15
PM_{10}	No wave	2.6 (0.8)	312	323	0.38	(reference)
	2	2.5 (0.8)	312	327	0.39	0.12
	3	2.7 (0.8)	308	327	0.42	0.03

From simple models of personal as a function of stationary exposure. * From ANOVA test of differences. ** adjusting for pollen season versus no pollen season (two-level indicator), *** adjusting for all three waves (three-level factor).
